# Exploring the Role of Dairy Consumption on the Growth and Development of Canadian Children: Protocol for a Longitudinal Mixed Method Research

**DOI:** 10.2196/87415

**Published:** 2026-04-08

**Authors:** Karim Karbin, Mojtaba Shafiee, Zoe L Longworth, Adam Baxter-Jones, Marta C Erlandson, Ginny Lane, Ling-Ling Tsao, Walter L Siqueira, Hassan Vatanparast

**Affiliations:** 1 College of Pharmacy and Nutrition, University of Saskatchewan, 107 Wiggins Road, Saskatoon, SK, Canada, 1-306-966-8866; 2 School of Public Health, University of Saskatchewan, Saskatoon, SK, Canada; 3 Margaret Ritchie School of Family and Consumer Sciences, College of Agricultural and Life Sciences, University of Idaho, Moscow, ID, United States; 4 College of Kinesiology, University of Saskatchewan, Saskatoon, SK, Canada; 5 College of Dentistry, University of Saskatchewan, Saskatoon, SK, Canada

**Keywords:** dairy consumption, child development, bone health, cognitive development, diet quality

## Abstract

**Background:**

Childhood is a critical period for physical and cognitive development, in which nutrition plays a fundamental role. Dairy products, rich in calcium, vitamin D, and high-quality protein, are essential for bone health, body composition development, and cognitive performance. However, evidence on the long-term impact of dairy consumption on children’s growth and development, particularly in the Canadian context, is limited. Additionally, recent revisions to the Canadian Food Guide, which classify dairy as part of the protein group rather than as a separate food group, may influence caregiver perceptions and children’s dairy intake.

**Objective:**

This protocol describes a study designed to examine the longitudinal relationship between dairy consumption and key indicators of growth, bone health, and cognitive development in Canadian children aged 5 to 11 years. The secondary objective is to assess caregivers’ and children’s knowledge, attitudes, practices, and perceived facilitators and barriers to dairy consumption.

**Methods:**

A longitudinal convergent parallel mixed methods study is being conducted with 4 data collection points over 2.5 years (baseline, and approximately 10, 20, and 30 months). Eligible participants include children aged 5 to 11 years at baseline, residing in the Saskatoon area, with no diagnosed conditions or medication use affecting bone metabolism or cognitive development. Quantitative data include dietary intake, anthropometrics, body composition, bone density and microarchitecture, subjective and objective measures of physical activity, cognitive assessments, and a salivary biomarker. The qualitative component aims to investigate facilitators and barriers to dairy consumption through interviews with caregivers. Mixed-effects models will be applied to assess longitudinal associations, and thematic analysis will be used to analyze qualitative data. Quantitative and qualitative findings will be integrated at the interpretation stage to inform overall conclusions.

**Results:**

Ethics approval was obtained from the University of Saskatchewan Research Ethics Board (Bio-3339) in 2022. Recruitment began in September 2022 and concluded in February 2025. A convenience sample of 166 volunteer children from Saskatoon was recruited, with efforts made to achieve demographic diversity. Data collection is ongoing and expected to finish by August 2027. Data analysis is underway, with preliminary findings expected to be published in 2026.

**Conclusions:**

This protocol outlines a comprehensive study that aims to generate context-specific evidence on the role of dairy in child development, with implications for developing responsive dietary guidance and public health policy in Canada.

## Introduction

Childhood is a period of rapid growth and development, during which a balanced diet is essential in supporting physical growth, cognitive development, and immune function [1]. Sufficient nutrient intake during this period helps maintain stable energy levels, supports bone and tooth development, and promotes healthy body composition [2]. In contrast, inadequate intake can restrict growth, impair cognitive performance, increase susceptibility to infections, and elevate the risk of obesity and chronic diseases later in life [2,3].

Dairy products are a rich source of nutrients such as high-quality protein, calcium, vitamin D, magnesium, and other minerals that are essential for skeletal development and overall growth [4,5]. Studies show that regular dairy consumption supports linear growth, influences body composition, and contributes to bone mineral accrual, potentially through growth factors such as insulin-like growth factor-1 (IGF-1) [6-8]. In many developed countries, dairy products are a major source of nutrients for children, supplying over 45% of calcium, 34% of iodine, 20% of potassium, 20%‐40% of vitamin B12, and 20% of vitamin A [4]. These nutrients are essential not only for growth and bone health but also for nerve function, muscle activity, and immune defense [9,10].

Emerging research considers dairy as a complex nutrient matrix (dairy matrix) where interactions between nutrients can improve bioavailability and produce synergistic effects, particularly regarding bone development [11,12]. Meta-analyses and randomized controlled trials have suggested that dairy consumption enhances bone mineral content (BMC) and bone mineral density (BMD), with the strongest effects observed among children with lower baseline calcium intake [6,7,13]. However, findings for body composition are inconsistent; some studies suggest associations with favorable weight outcomes, while systematic reviews report largely nonsignificant effects on lean or fat mass [14-16]. Preliminary evidence has also linked dairy intake with cognitive outcomes, such as executive function [17], but most available evidence is from cross-sectional data, which limits causal interpretation and complicates distinguishing dietary effects from normal maturation processes [4].

Growth during childhood is influenced by more than age and nutrition. Biological maturation, which varies widely among children, affects growth rate, bone development, and body composition [18]. Without accounting for maturation status, it becomes challenging to determine whether observed differences in growth are attributable to nutrition or developmental timing.

In the Canadian context, national dietary trends show declining dairy intake among children, coinciding with increased use of plant-based alternatives that differ in nutrient density and bioavailability [19,20]. At the same time, Canada’s 2019 Food Guide repositioned dairy into a broader “protein foods” category, reducing its distinct positioning in national dietary recommendations [21]. While intended to promote dietary variety, sustainability, and ultimately to support optimal public health, this change may unintentionally downplay the distinct nutritional contributions of dairy, particularly for children [22]. Because caregivers’ perceptions and eating behaviors strongly shape children’s dietary practices, this shift could lead some caregivers to view dairy as interchangeable with other protein sources, potentially reducing children’s intake and influencing child health outcomes [12]. However, these behavioral and perceptual dimensions remain largely unexplored.

This study is informed by life course theory and a systems-oriented perspective to investigate child nutrition. Life course theory identifies childhood as a critical period during which dietary exposures, such as milk and dairy intake, may affect growth, bone health, and long-term health outcomes [23]. In addition, from the perspective of the socioecological framework of health behavior, this study positions children’s dairy consumption as part of a network of influences shaped by both individual nutritional requirements and caregiver practices influenced by environmental and cultural factors [24].

Guided by these frameworks, the study examines multiple domains of child development, including growth, body composition, bone density and microarchitecture, and cognitive function, while accounting for dietary intake, physical activity, biological maturation, and sociodemographic factors. In parallel, qualitative assessments from caregivers capture modifiable behavioral and contextual determinants of dairy intake, such as perceived risks and benefits and barriers and facilitators toward dairy consumption and perceptions of dietary guidance.

So far, limited research has explored how dairy intake connects to various aspects of growth and development in school-aged children [17,25], as well as how caregiver perceptions might affect these results. To address these gaps, this study will use a longitudinal mixed methods design to track dairy consumption and developmental indicators among Canadian children aged 5 to 11 years. This approach allows for the modeling of individual growth trajectories and helps distinguish nutrient-specific effects from age-related changes, such as biological maturation. By following children over time, the study aims to strengthen causal inference and provide more detailed insight into how dairy intake may influence physical and cognitive development. The findings will contribute to evidence-based dietary guidance and public health strategies that support optimal growth and development in school-aged children.

## Methods

### Study Design

**Figure 1.**
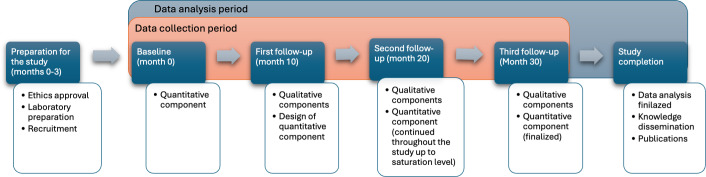
Timelines of the study.

This study uses a longitudinal convergent parallel mixed methods design with both quantitative and qualitative components. The longitudinal framework captures developmental changes and the cumulative effects of diet over time. Quantitative measures include dietary intake, growth trajectories, bone health and body composition parameters, cognitive assessments, and a structured knowledge, attitudes, and practices (KAP) questionnaire, allowing statistical evaluation of how dairy intake and dairy perceptions relate to developmental outcomes. The qualitative component explores facilitators and barriers to dairy consumption among caregivers. Participants will be followed for 2.5 years, with assessments at 4 points approximately 10 months apart. Figure 1 presents the study timeline. This study protocol is reported in accordance with the ObsQual Protocol Checklists, a structured guidance tool for observational study protocols [26]. The checklist is available in Multimedia Appendix 1.

### Study Population and Sample Size

Children aged 5‐11 years were selected as the target population to capture a developmental stage characterized by steady linear growth, rapid bone mineral accrual, and meaningful cognitive advancement before the onset of puberty [2,27]. During this middle childhood period, biological maturation is variable but less pronounced than in adolescence, allowing clearer modeling of nutritional influences without the confounding effects of pubertal growth surges [28 ]. This age range also represents a stage in which caregivers continue to exert substantial control over children’s dietary behaviors, making it an optimal window for assessing both dietary exposures and caregiver-related influences [29 ].

The target population for this study consists of children aged 5‐11 years residing in Saskatoon, Canada. According to the 2016 Canadian census, Saskatoon had a total of 44,305 children aged 0 to 14 years, with 14,975 in the 5- to 9-year age group and 13,705 in the 10- to 14-year age group. The total child population consisted of 14,765 (51.5%) boys and 13,915 (48.5%) girls and included 6160 immigrant children [30].

The study sample size was determined to ensure sufficient statistical power to detect a clinically meaningful relationship between dairy intake and linear growth in children aged 5 to 11 years. Linear growth, defined as the change in height over time, served as the primary outcome. The study used a repeated-measures design with 4 data collection points at baseline, 10, 20, and 30 months. Based on prior literature and expert input, the analysis assumed a 2-sided α of .05% and 80% power. The residual SD of height was estimated at 0.5 cm, and the intrasubject correlation was set at 0.8, reflecting expected consistency in individual height measurements over time [31]. The SD for daily dairy intake was approximated at 1 serving per day [32,33]. A clinically meaningful effect was defined as an additional 0.2 cm of height gain over 30 months per extra daily serving of dairy, corresponding to approximately 0.0067 cm per month per serving. This estimate was informed by meta-analytic findings, suggesting a 0.4 cm per year increase in height associated with an additional 245 mL of milk consumed daily [34]. Using a power analysis approximation for a linear mixed-effects model that incorporates the effect of a continuous covariate (dairy intake) on growth rate, the required sample size was estimated at 87 participants. To account for an anticipated 25% attrition over the 30-month study period, the recruitment target was increased to 116 participants.

A combination of convenience and snowball sampling methods was used to recruit participants, reflecting the demographic characteristics of children in Saskatoon. Recruitment was monitored to maintain a balance between boys and girls, and demographic variables such as age and newcomer status were tracked to ensure proportional representation approximating the actual population. Inclusion criteria are (1) children aged 5 to 11 years, (2) healthy status (as determined by parental report and study screening), and (3) no current treatment for malnutrition or other serious medical conditions. Exclusion criteria include (1) chronic diseases or medical conditions affecting growth and development, (2) chronic medication use, and (3) medications known to affect bone metabolism or calcium balance. Retention efforts will rely on flexible scheduling, reminders, and small incentives.

### Study Setting

The study is being conducted in Saskatoon, Canada, leveraging various recruiting channels, including libraries, recreational and community centers, social media, and online platforms, to ensure a diverse and representative sample. Assessments are being conducted in research facilities at the University of Saskatchewan.

### Ethical Considerations

Ethics approval has been obtained from the University of Saskatchewan Ethics Board (Bio-3339). Written informed consent was obtained from the parents or legal guardians of all participating children. During the consent process, parents or guardians received detailed information about the study’s purpose, procedures, potential risks, and benefits and had the opportunity to ask questions before signing the consent form. Age-appropriate assent was also obtained from each child to ensure understanding and voluntary participation. Children were informed that they could withdraw from the study at any time without consequence. To protect participant privacy and confidentiality, all data are stored on secure, access‑restricted servers housed at the University of Saskatchewan. Identifiable information is maintained separately from study data and replaced with unique coded identifiers. Only authorized members of the research team have access to the linkage file. Any published results will present aggregated findings only, ensuring that no individual participant can be identified. Parents and participating children will receive 1 gift card per follow-up visit as compensation, provided in accordance with institutional guidelines. Compensation is distributed uniformly to all participants and is not dependent on full study completion, which minimizes the risk of coercion or undue influence.

### Data Collection

#### Overview

Data are collected at months 0, 10, 20, and 30 using both quantitative and qualitative methods outlined in Table 1. These repeated measurements allow the study to track changes over time and capture comprehensive information on the participants.

**Table 1.** Data collection instruments and time points.

Type of data and variable	Instrument or method	Month 0	Month 10	Month 20	Month 30
Contact information and consent	Caregiver consent form and contact information form	✓			
Quantitative					
Sociodemographic and socioeconomic status	Questionnaire (caregiver-reported)	✓			
Food security questionnaire	HFSSM^a^	✓			
Anthropometric measures	Standardized measurement protocol	✓	✓	✓	✓
Dietary intake	Multiple 24-hour dietary recalls (interviewer-administered)	✓	✓	✓	✓
Habitual dairy intake	Dairy Food Frequency Questionnaire	✓	✓	✓	✓
Physical activity (self-report)	PAQ-C^b^	✓	✓	✓	✓
Physical activity (Objective)	Accelerometers		✓		✓
Sun exposure	Sun exposure questionnaire	✓	✓	✓	✓
Skin pigmentation	Konica Minolta spectrophotometer	✓			✓
Body composition and bone health	DXA^c^	✓	✓	✓	✓
Bone microarchitecture and density	HR-pQCT^d^	✓	✓		
Biomarker of growth and development	Saliva sample for IGF-1^e^ analysis	✓		✓	✓
Cognitive function	KABC-II^f^		✓		✓
Child dairy knowledge, attitudes, and practices toward dairy product consumption	KAP^g^ survey		✓		✓
Caregiver knowledge, attitudes, and practices toward dairy product consumption	KAP survey		✓		
Qualitative					
Facilitators and barriers influencing child dairy consumption	Semistructured interview with caregivers		✓		

a HFSSM: Household Food Security Survey Module.

b PAQ-C: Physical Activity Questionnaire for Children.

c DXA: dual-energy x-ray absorptiometry.

d HR-pQCT: high-resolution peripheral quantitative computed tomography.

e IGF-1: insulin-like growth factor 1.

f KABC-II: Kaufman Assessment Battery for Children, Second Edition.

g KAP: knowledge, attitudes, and practices.

#### Quantitative Component

##### Overview

We assess dietary intake, physical activity, anthropometrics, body composition (including bone, fat, and muscle mass), cognitive performance, and a salivary biomarker of growth and development. At baseline, we also collect sociodemographic and socioeconomic data using standardized questionnaires from the Canadian Community Health Survey (CCHS) [35]. These instruments record age, sex, ethnicity, household income, education, source of income, immigration status, and duration of residence in Canada. Using the CCHS format allows us to compare our sample with national population data, strengthening the generalizability of the results.

##### Dietary Assessment

To obtain a reliable estimate of individual dietary intake, a combination of serial 24-hour dietary recalls and a dairy-specific Food Frequency Questionnaire (FFQ) is administered at each of the 4 data collection time points [36,37]. Three 24-hour dietary recalls are scheduled approximately 10 to 14 days apart and on different days of the week to capture day-to-day variation. These recalls follow the multiple-pass method, with the primary caregiver reporting as a proxy to ensure accuracy [38,39]. The mean of the three 24-hour recalls at each time point provides an estimate of usual dietary intake, and the ESHA Food Processor (version 11.6.522; ESHA Research) is used to determine the nutrient composition of the foods. The dairy FFQ, which assesses the frequency of dairy consumption over the previous 6 months, is adopted from the Canadian Health Measures Survey Food Frequency Questions that are specific to milk and dairy products and plant-based dairy alternatives. This questionnaire comprehensively assesses the dairy food group compared to 24-hour recall data at the national level [40].

##### Food Security

At baseline, food security status is being assessed using the US Department of Agriculture Household Food Security Survey Module [41] and interpreted using the PROOF guide for measurement in Canadian contexts [42]. The Household Food Security Survey Module contains 18 questions about household food security over the past 12 months. Ten of the items are specific to the experiences of adults in the household, and the remaining 8 items are specific to the experiences of children (<18 years). Responses are scored in accordance with US Department of Agriculture item-affirmative criteria, and food security categories (food secure, marginal, moderate, and severe) are assigned using thresholds adapted per the PROOF guide [42]. Both household-level and child-level food insecurity are derived from the corresponding adult and child items. This assessment will allow an analysis of the potential role of food security status in food choices, particularly dairy foods.

##### Anthropometric Measurements

Anthropometric data are being collected at all time points following standardized protocols. Standing height is measured to the nearest 0.1 cm using a wall-mounted stadiometer. Participants stand with their shoes off, heels against the stadiometer, arms by their sides, and eyes aligned with the Frankfurt horizontal plane, taking a deep breath [43]. Sitting height is measured from the head’s vertex to the seat surface and will be used to estimate age at peak height velocity (PHV), a proxy for somatic maturity status. PHV reflects the timing of the adolescent growth spurt and is a widely used noninvasive indicator of biological maturity in children [44]. Body weight is recorded to the nearest 0.01 kg using a calibrated SECA electronic scale, with participants wearing light indoor clothing and no footwear or jewelry [45]. An average of 3 measurements taken for all anthropometric variables is used to minimize random measurement error [45].

##### Body Composition

Body composition, including lean mass, fat mass, and percent body fat, is measured using dual-energy x-ray absorptiometry (DXA; Hologic Discovery-Wi) at each assessment time point. DXA is the gold standard method for evaluating body composition in children and adolescents, given its minimal radiation exposure and noninvasive nature [46]. All scans are performed by certified radiology technicians following the manufacturer’s recommended calibration and positioning procedures.

##### Bone Health Assessments

###### Dual-Energy X-Ray Absorptiometry

BMC and areal BMD are measured using DXA at the total body, hip, and lumbar spine during each of the 4 data collection time points. DXA provides highly reproducible estimates of areal BMD and BMC, enabling the evaluation of skeletal mass and mineralization [46].

###### High-Resolution Peripheral Quantitative Computed Tomography

Bone microarchitecture and compartment-specific volumetric BMD are being assessed with high-resolution peripheral quantitative computed tomography (HR-pQCT) at the distal radius and tibia. This low-radiation, noninvasive technique generates high-resolution 3D images of trabecular and cortical compartments, allowing detailed analysis of volumetric BMD, bone geometry, and microstructural integrity, which are important indicators of bone strength and fracture risk [47].

### Physical Activity Assessment

Physical activity is measured using both subjective and objective methods. For subjective assessment, the Physical Activity of Children and Youth module from the CCHS is administered [48]. This module forms part of the health components within the CCHS and collects information on physical activity behaviors among children and youths. It is used nationally to monitor movement behaviors and complements other health and nutrition data collected through the survey. To complement self-reported data, accelerometers are used to provide objective measures of physical activity. Worn on the hip for 7 consecutive days, these devices record continuous data on movement patterns, including time spent in sedentary, light, moderate, and vigorous activity levels.

### Skin Tone and Sun Exposure

Using the related Canadian Health Measures Survey, outdoor activity and duration of sun exposure are measured based on the number of hours and the period of the day. Further, because the study includes children from different ethnic backgrounds with varying skin pigmentation (important for UV absorption and vitamin D synthesis), a compact portable spectrophotometer (CM-600d, Konica Minolta) is used with skin analysis software to measure skin pigmentation [49].

### Salivary Biomarker of Growth and Development

In this study, salivary IGF-1 is examined as a noninvasive biomarker of growth and development in children [50]. Saliva samples are collected using the Oasis Pure•SAL-C Kit between 1 PM and 4 PM to maintain uniform conditions and minimize diurnal variation in IGF-1 levels. The collected saliva samples are analyzed using enzyme-linked immunosorbent assay techniques. Enzyme-linked immunosorbent assay is a highly sensitive and specific method that allows for the quantification of IGF-1 concentrations in biological samples [51].

### Cognitive Assessments

Cognitive functioning in children is assessed using the Kaufman Assessment Battery for Children, Second Edition (KABC-II), a standardized, individually administered instrument designed to evaluate a broad range of cognitive abilities in children aged 3 to 18 years [52]. The KABC-II is grounded in contemporary theories of intelligence, including the Cattell-Horn-Carroll model and Luria’s neuropsychological framework, making it particularly appropriate for use in diverse populations [53]. It provides composite scores across key cognitive domains, including sequential processing, simultaneous processing, learning, planning, and knowledge, as well as an overall Fluid-Crystallized Index [53]. In this study, the KABC-II is administered to children at 2 time points (months 10 and 30). The assessment typically requires 90 to 150 minutes to complete and is conducted in a quiet, child-friendly environment by trained research personnel. Administration and scoring follow standardized procedures outlined in the KABC-II manual [53].

### KAP Survey

Two separate KAP questionnaires on dairy foods were developed for caregivers and children following the Food and Agriculture Organization guidelines for assessing nutrition-related KAP [54] and grounded in the health belief model [55]. The caregiver questionnaire is administered once, at month 10, to capture baseline KAP regarding dairy consumption, while the child questionnaire is administered at 2 time points (months 10 and 30) to assess changes over time. Both questionnaires were developed by the research team and reviewed for content relevance, clarity, and age appropriateness. In combination with children’s dietary intake data, these questionnaires capture KAP regarding dairy foods within participating families.

### Qualitative Component

The qualitative component of the study consists of semistructured interviews with a subset of primary caregivers involved in the study. The interviews explore facilitators and barriers to milk and dairy product consumption. Participants are selected from the overall study sample using purposive sampling to ensure representation across ethnic and socioeconomic groups, with interviews continuing until data saturation is achieved [56,57]. Interviews are conducted in private, face-to-face settings convenient for caregivers, and are audio-recorded. Each interview lasts about 30 minutes with informed consent.

### Data Management

All data are managed in accordance with the project’s Data Management Plan and institutional ethics protocols, ensuring confidentiality, data integrity, and controlled access for authorized personnel only. Researchers may request access to nonrestricted anonymized data by submitting a proposal to the principal investigator (HV). As this is a protocol, no data currently exist.

Quantitative data are captured using REDCap (Research Electronic Data Capture; Vanderbilt University), a secure web-based system. Quantitative data from field and laboratory measurements (eg, dietary recalls, anthropometry, sociodemographics, food security, salivary IGF-1, DXA, and HR-pQCT) are entered directly into REDCap. Additional quantitative datasets, such as KABC-II results and accelerometry outputs, are exported from specialized software as digital or CSV files and stored securely in the institutional repository. Dietary recalls data are coded with the ESHA Food Processor software and exported as CSV files. Qualitative data, including audio-recorded interviews and REDCap interview notes, are transcribed, anonymized, and structured, then imported into NVivo (QSR International) for analysis, with the original audio files archived in secure formats.

### Quality Assurance and Monitoring

The study incorporates a comprehensive quality assurance and monitoring strategy to ensure the collection of high-quality and reliable data. A foundational element of this approach is the rigorous training of research assistants (RAs). All RAs participate in training sessions that include an overview of the study protocol, ethical procedures, data collection methods, as well as instruction in using REDCap for data entry and NVivo for qualitative data analysis. The training combines theoretical instruction with hands-on demonstrations to build competency, with refresher sessions held periodically to reinforce protocol adherence and address emerging challenges.

Ongoing supervision and regular data audits are conducted to uphold data integrity throughout the study. Supervisors perform routine checks on field activities to ensure compliance with research protocols and provide immediate, constructive feedback to RAs. Randomly selected data entries are audited for accuracy, completeness, and consistency. These audits include a thorough examination of missing values, outlier handling, and the calculation of derived variables. Any discrepancies identified are promptly corrected and documented, ensuring transparency and continued quality control.

### Outcomes

The primary outcome of this study is the association between dairy consumption and bone and body composition outcomes in children aged 5 to 11 years. Bone outcomes are assessed using DXA, including BMC and BMD at the lumbar spine, femur, and total body, as well as lumbar spine bone mineral apparent density. Body composition outcomes are also derived from DXA and include total body muscle mass, fat mass, and percent body fat. These measures are used to describe how dairy intake may be linked to skeletal development and overall body composition during middle childhood.

Secondary outcomes include broader indicators of growth and development. The study evaluates how milk and dairy foods contribute to total dietary intake and overall diet quality. It also examines associations between dairy consumption and cognitive development, as well as hormones and biomarkers connected to growth, including IGF-1. These outcomes help clarify how dairy intake may relate to developmental processes beyond bone health.

The study also investigates KAP related to dairy consumption among children and their caregivers. This includes an assessment of factors that influence dairy intake over time, such as household routines, access to foods, and perceived supports or challenges. Quantitative measures are used to identify facilitators and barriers to dairy consumption among Canadian children. Together, these outcomes provide a detailed picture of how dairy intake fits within patterns of growth, development, dietary behavior, and family context. By addressing these primary and secondary outcomes, the study aims to provide comprehensive insights into the role of dairy consumption in the growth, development, and overall health of Canadian children. The findings will contribute to evidence-based dietary recommendations and interventions aimed at improving child health outcomes.

### Data Analysis Plan

The research uses a mixed methods approach, using both quantitative and qualitative data. Quantitative data analyses follow the internal data processing protocol, beginning with data cleaning and preparation. This process includes checking for data entry errors, examining distributions, and identifying missing data and outliers. Data entry issues are evaluated by inspecting frequencies, identifying misspelled entries for categorical variables, and examining minimum and maximum values, means, histograms, and ranges for continuous variables. Loss to follow-up will be addressed by comparing baseline characteristics of participants who complete the study with those who withdraw. These comparisons will draw on standardized mean differences and logistic regression to identify differences in demographics, dairy intake, and growth measures [58]. Missing data are addressed using multiple imputation or maximum likelihood methods, depending on the pattern of missingness [59].

Once cleaned, the dataset is used to generate derived variables relevant to the study objectives. These include anthropometric indicators such as BMI, BMI *z* scores, height-for-age, and weight-for-age *z* scores; dietary measures such as total dairy intake, calcium intake, and diet quality indices; and consumption groupings based on children’s dairy intake relative to national dietary guidelines. Descriptive statistics summarize participant characteristics and key outcomes. Continuous variables are reported as means with SDs or as medians with IQRs, and categorical variables are summarized as counts and percentages.

Longitudinal associations between dairy intake and outcomes such as somatic growth, body composition, bone health, and cognitive development are evaluated using mixed-effects models and generalized estimating equations. Mixed-effects models account for repeated measures within participants and include random intercepts and slopes where appropriate [60]. Generalized estimating equation estimates population-averaged effects while accounting for within-subject correlation over time [60]. Covariates, including age, sex, socioeconomic status, physical activity, and total energy intake, are incorporated to control for potential confounding. Subgroup analyses will be conducted for male and female participants separately to explore sex-specific associations and developmental trajectories. Interaction effects by sex and baseline diet quality are also examined. Sensitivity analyses assess the robustness of findings, including alternative categorizations of dairy intake and different approaches to handling missing data. Exploratory analyses may be used to examine associations between diet quality indices and secondary outcomes such as bone microarchitecture using regression or correlation models. All quantitative analyses are performed using R (R Foundation for Statistical Computing) or SPSS (IBM Corp) software, in accordance with internal protocols to ensure reproducibility and transparency.

Qualitative data from caregiver interviews and child surveys are analyzed using inductive thematic content analysis. The analysis begins with familiarization through transcript review, followed by iterative coding using NVivo software. Codes are grouped into preliminary themes, which are then reviewed and refined. To ensure consistency and reduce bias, a second coder independently codes approximately 15% of the data using a predefined codebook. Intercoder agreement is quantified using Cohen κ, and any discrepancies are resolved through discussion. Thematic saturation is monitored throughout, and analysis continues until saturation is achieved.

### Knowledge Translation

The study uses a comprehensive knowledge translation strategy involving strong collaboration with key partners such as Osteoporosis Canada, public health agencies, community-based child health organizations, and immigrant and cultural health advocacy groups. A dedicated working group will coordinate stakeholder engagement and tailor dissemination strategies for specific audiences, including the public, service providers, policymakers, and academics. Efforts will prioritize cultural sensitivity, particularly for immigrant and Indigenous communities. Dissemination will include policy briefs, workshops, peer-reviewed publications, and conference presentations. Continuous evaluation will guide adaptive improvements to ensure broad impact and sustained use.

## Results

Recruitment began in September 2022 and concluded in February 2025, following ethics approval by the University of Saskatchewan Research Ethics Board (Bio‑3339) in October 2022. A total of 116 children and their primary caregivers were enrolled. Participants are being followed across 4 visits spaced approximately 10 months apart. Retention rates have remained strong, with 102 participants completing the second visit, 47 completing the third visit, and 25 completing the fourth visit to date. Data collection is ongoing and is expected to continue through August 2027. Preliminary study findings are expected to be published beginning in fall 2026, with initial descriptive results on dietary patterns and KAP, providing early insights into dairy consumption behaviors among the study population.

## Discussion

To the best of our knowledge, this is the first study in Canada investigating the longitudinal relationship between dairy consumption and child growth, bone health, and cognitive development using a mixed methods design. This study is expected to clarify how dairy intake relates to child growth, bone health, and cognitive development. It also considers the social and behavioral factors that shape dietary patterns. The design brings together quantitative measures of dietary intake, anthropometry, body composition, bone health, and cognitive assessments, with qualitative insights from caregivers. Taken together, the mixed methods design allows the study to describe both the biological outcomes and the circumstances that influence how children consume dairy foods.

While the majority of prior research on dairy intake in children originates from cross-sectional studies [4], this longitudinal study enables the assessment of patterns across multiple time points, providing a more comprehensive understanding of the evolution of dietary intake and growth and development outcomes. This approach addresses the knowledge gap in the longitudinal studies. For example, the Iowa Fluoride Study in the United States found that consistent milk consumption from age 2 to 17 years was associated with a small increase in adult height, specifically 0.39 cm for each extra 8-ounce daily serving [61]. The Generation R Study in the Netherlands showed that adherence to a “dairy and whole grains” pattern was positively associated with BMD and area-adjusted BMC in children aged 8 to 10 years [62]. In China, a randomized trial in preschool children reported an increase in arms BMD and BMC with milk supplementation [6]. However, few studies have followed children over time while also considering how caregivers shape food choices. By focusing on Canadian children aged between 5 and 11 years, this study captures a period of consistent growth and bone development before the onset of maturation complicates nutritional models. It also extends findings from US preschool cohorts, in which children who consumed more milk were taller but also showed higher BMI. Validated tools ensure data quality, including DXA/HR-pQCT for bone, multiple 24-hour recalls or FFQs for diet, and standardized cognitive assessments [36,37,39,52].

Despite its strengths, the study has potential limitations that could affect the validity of its findings. Selection bias could occur if participants are healthier or of higher socioeconomic status than the general population; this risk is mitigated through targeted recruitment weighted against census data [58,63]. Measurement bias is also a concern, particularly in dietary recalls or anthropometric assessments [64]. For example, 24-hour dietary recalls in children underestimate intake by 15%‐24% due to omissions, especially of snacks, as validated against observational methods [65]. To minimize these risks, rigorous training for research staff, standardized data collection protocols, and validated dietary assessment tools have been implemented. Differences in age and maturity, such as early puberty in girls influencing growth and bone development, are addressed by controlling for age at PHV [66]. Residual confounding, attrition, and dairy heterogeneity (eg, milk vs yogurt) remain challenges, which can be addressed using mixed-effects models and sensitivity analyses.

The extent to which findings from this study can inform broader Canadian dietary guidance also needs to be considered. While CCHS data show that suboptimal adherence to dietary recommendations is common across regions [67], Saskatoon represents a mid-sized Prairie urban context with distinct demographic, socioeconomic, and cultural characteristics that may shape dairy intake behaviors [68]. Therefore, the study population should be viewed as illustrative rather than fully representative of all Canadian children. Nevertheless, insights into longitudinal dietary patterns, developmental outcomes, and caregiver-reported barriers provide meaningful evidence on mechanisms relevant to national policy, particularly when interpreted alongside nationally representative data.

Overall, this study provides evidence on the effects of dairy consumption on child development and identifies facilitators and barriers to dairy intake from the perspectives of both caregivers and children. The findings are expected to inform responsive dietary guidance, influence public health policy, and guide future interventions to support optimal child nutrition and development.

## Supplementary material

10.2196/87415 Multimedia Appendix 1 Facilitators and barriers to dairy product consumption—interview guide.

10.2196/87415 Peer Review Report 1 Peer review report by the Dairy Farmers of Canada.

## Abbreviations

BMC: bone mineral content
BMD: bone mineral density
CCHS: Canadian Community Health Survey
DXA: dual-energy x-ray absorptiometry
FFQ: Food Frequency Questionnaire
HR-pQCT: high-resolution peripheral quantitative computed tomography
IGF-1: insulin-like growth factor-1
KABC-II: Kaufman Assessment Battery for Children, Second Edition
KAP: knowledge, attitudes, and practices
PHV: peak height velocity
RA: research assistant
REDCap: Research Electronic Data Capture
