# Supplementary material for: Exploring the Role of Dairy Consumption on the Growth and Development of Canadian Children: Protocol for a Longitudinal Mixed Method Research
Source: JMIR Res Protoc. 2026 Apr 8;15:e87415. doi: 10.2196/87415 (PMC13060745; doi:10.2196/87415)
Supplement: Multimedia Appendix 1 [file resprot-v15-e87415-s001.docx]

Sources:

Laila, A., Topakas, N., Farr, E., Haines, J., Ma, D. W., Newton, G., Buchholz, A. C. Barriers and facilitators of household provisions of dairy and plant-based diary alternatives in families with preschool-age children. *Public Health Nutrition: 24*(17), 5673 -5685.

Racey, M., Bransfield, J., Capello, K., Field, D., Kulak, V., Machmueller, D., Preyde, M., Newton, G. Barriers and facilitators to intake of dairy products in adolescent males and females with different levels of habitual intake. *Global Pediatric Health: 4*(2017).

These questions are intended to guide the semi-structured interview process with participants caregivers. Research personnel will be trained in interview techniques.

1. When I say ‘dairy products’ what do you think of?
2. What are the benefits of your child consuming dairy products?

*Probe – If participant explains benefit: What about dairy products make them healthy?

1. Are there any risks to your child for consuming dairy products?

*Probe – If participant explains risks: What about dairy products make them unhealthy? Where did you get this information?

1. Are dairy products available in your house for your children to consume?
2. Are there any other factors which may motivate you to offer dairy products to your child?

*Probe – specific meals, locations, packaging

1. Are there any other factors which may stop you from offering dairy products to your child?

*Probe – specific meals, locations, packaging

1. When and where does your child consume dairy products?

*Probe – for meals throughout the day (i.e., breakfast, snack, lunch, dinner, dessert) and various locations (i.e., school, daycare, friends’ and relatives’ houses, restaurants, etc.)

1. What kinds of dairy products does your child consume at home? What about outside of the home?

* Probe – for type of dairy products (i.e., milk, yogurt, cheese, ice cream, alternatives or substitutes such as almond/soy milk, coconut yogurt, etc.)

1. How do you determine whether your child is consuming enough or not enough dairy products?
2. Do you know what the current recommendations for child dairy product consumption in Canada are? If so, explain. [If the participant does not know, provide explanation of recommendations]

*Probe – what is your opinion of these recommendations? Do you agree/disagree? Why?

1. What circumstances make it easy for you to offer dairy products to your child?

*Probe – specific meals, locations, packaging

1. What circumstances make it more difficult for you to offer dairy products to your child?

*Probe – specific meals, locations, packaging

1. When shopping, what do you look for in dairy products?

*Probe – cost, convenience, accessibility, availability, perishability, fat content, salt content, sugar content

1. What would motivate you to increase how many dairy products you offer your child?

*Probe – friends, convenience, knowledge about health

1. Has the COVID-19 pandemic impacted your dairy consumption patterns in your household?

*Probe – how? Is it a result of cost/ food prices? Accessibility?

1. Is there anything else you want to add?
