# Supplementary material for: Exploring the Role of Dairy Consumption on the Growth and Development of Canadian Children: Protocol for a Longitudinal Mixed Method Research
Source: JMIR Res Protoc. 2026 Apr 8;15:e87415. doi: 10.2196/87415 (PMC13060745; doi:10.2196/87415)
Supplement: Peer Review Report 1 [file resprot-v15-e87415-s002.pdf]

DAIRY FARMERS OF CANADA
LES PRODUCTEURS LAITIERS DU CANADA

November 17, 2021

Hassan Vatanparast, Professor
University of Saskatchewan
vatan.h@usask.ca

Dear Dr. Vatanparast,

We are pleased to inform you that your research project entitled: **"***The role of dairy food consumption in optimal growth and development of Canadian children: A longitudinal mixed-method study"* has been accepted for funding as part of Dairy Farmers of Canada (DFC)'s Special Call for Proposals in Optimal Growth and Development in Children and in Dairy Protein, for a **total amount of $399,852.**

Please send us a **revised proposal** that takes into account the following comments that you addressed:

- Need to see a better power calculation - prevalence of exposure and delta incorporated into this calculation, number of participants, etc.
- Might want to consider a different primary outcome.
- Some concerns with sample size calculation based on height.

Please also find enclosed the comments of the reviewers for your consideration.

Prior to the initiation of the project, a Memorandum of Agreement must be signed by and between the research institution, the principal investigator and DFC. Please provide **Natalie Morin** (natalie.morin@dfc-plc.ca) the name and the email of the person in charge of the research agreements in your institution as soon as possible.

We are looking forward to working with you and to the successful completion of your project.

Sincerely,

Maria Kalergis

Maria Kalergis, PhD, RD, CDE
National Program Manager, Nutrition - Scientific Affairs

MK/nm

The External Reviewer's Report should include:

1. An assessment of the Principal Investigator

Comment particularly on the TRAINING and RESEARCH EXPERIENCE.
(For those applying for a renewal of grant, comment on the relevant RESEARCH EXPERIENCE, SIGNIFICANT CONTRIBUTIONS to the field, the PRODUCTIVITY (i.e. publications) during the present period of funding.)

The principal investigator (PI) is a distinguished scientist who has extensive experience conducting longitudinal studies including the internationally recognized and highly successful, "Pediatric Bone Mineral Study." He has published over 150 peer-review publications and has a very good h-index of 34. Currently, he is principal investigator or co-investigator on grants totalling over 4 million dollars. His expertise in nutrition and growth epidemiology, especially related to nutrition components of dairy foods, is ideal for the proposed project. Moreover, Dr. Vatanparast has a strong history of collaboration with the co-investigators.

2. A synopsis of the proposal

<u>For Operating Grants:</u> Identify the PURPOSE of the proposal, the HYPOTHESIS to be tested, or the QUESTIONS to be answered, the OBJECTIVES to be achieved, the APPROACH proposed, and the PROGRESS made to date.

<u>For Special Instrumentation or Equipment Requests:</u> Comment on the proposed uses, appropriateness and availability of similar equipment or services to the applicant's laboratory.

The investigators will use a "contextually longitudinal explanatory sequential mixed-method design with quantitative and qualitative components." The primary objective of the study is to determine how dairy consumption is associated with growth and development indicators, including cognitive development, of Canadian boys and girls aged 5-11 years over a 2.5-year period. Another objective is to determine knowledge, attitude and practice of dairy products consumption, as well as facilitators and barriers to dairy consumption, among Canadian children over time. Children, 5-11 years of age will be recruited and followed for 2.5 years with measurements every six months for most outcome measures. The research team hypothesizes that "appropriate intake of milk and dairy products has a positive association with growth and development parameters in these children."

3. An assessment of the proposal

Discuss the strengths and weaknesses of the project. Assess the scientific merit

of potential of the work proposed. State your recommendation, give clear and concise justification.

Strengths

Research team - the multidisciplinary research team is a major strength of the proposed project. Dr. Vatanparast's analytical skills with longitudinal studies, Dr. Baxter Jones' expertise with childhood growth, especially his experiences with the long-term "Pediatric Bone Mineral Study," and Dr. Haines' experience and knowledge of diet and nutrition methodologies and in particular, dairy foods, make this multidisciplinary research team ideal to address the objectives of the Special Call. Furthermore, high manuscript productivity and translation of the knowledge gained to the Canadian public, would be expected from this group of scientists.

Study design - while the gold standard study design, a randomized, controlled trial, is the favored study design in this Special Call, the longitudinal study design proposed by the investigators should provide more valuable and unique data on dairy food consumption and growth in young children. With a study duration of 2.5 years and 6 measurement timepoints, the investigators should be able to successfully capture growth outcomes, typical intakes of dairy foods and barriers and facilitators to dairy consumption. Inclusion of newcomer participants into the study will provide useful information about dairy food intakes and facilitators and barriers to consumption in diverse populations. This is critical with the increasing numbers of newcomers to Canada.

Methods - The proposed methods, another major strength of the project, are scientifically sound. The investigators have employed the methods extensively in longitudinal studies. With respect to milk and dairy food consumption, the combined use of serial 24-hour recalls and the Canadian health Measures Survey Food Frequency Questionnaire specific to milk and dairy products, should provide accurate and reliable intakes for milk and dairy foods over the 2.5-year timeframe. The use of Dual Energy X-Ray Absorptiometry for body composition assessment is the gold standard and the research team member, Dr. Erlandson, has extensive experience in this aspect of the study. Importantly, the PI will use the quantitative data to inform the qualitative approach and the qualitative analysis will be used to inform the modeling of the quantitative analyses and interpretation of the quantitative data.

Weaknesses

There were few weaknesses in the proposal and they are minor:

Under the experimental approach, the order of the specific objectives should align with the overarching goal of the study (i.e., objective 1 should address growth).

In the background section, the rationale for inclusion of the cognitive component of the study could have been more compelling.

Quality-control procedures should have been described for many of the methods including bone and body composition, skin tone and sun exposure, salivary IGF-I, and cognition. In addition, quality-control issues for longitudinal studies should have been addressed in the experimental design section.

Recommendation

The proposed project is an innovative approach that will comprehensively address the objectives of the Special Call, “the role of dairy in the optimal growth and development of children and the role of dairy protein foods within a healthy plant-based diet and especially in relation to plant-based protein foods. For the purposes of the Special Call, I highly recommend the proposed project for the study strengths stated above.

The External Reviewer's Report should include:

1. An assessment of the Principal Investigator

Comment particularly on the TRAINING and RESEARCH EXPERIENCE.
(For those applying for a renewal of grant, comment on the relevant RESEARCH EXPERIENCE, SIGNIFICANT CONTRIBUTIONS to the field, the PRODUCTIVITY (i.e. publications) during the present period of funding.)

The applicant appears to be well qualified and to have consistently attracted competitive research funding. He appears to currently (2021) hold $592.5k as PI and is involved in $3,720k as a co-applicant. Some of these studies relate to nutrition and health in children but the majority appear to focus on adult samples.

Dr. Vatanparast is a full professor at a well-recognised Canadian university with a strong track record for research in child health and growth. In the last two years (2020-2021) he appears to have been involved in 24 publications: 2 as lead author, 10 as senior author and 12 as a co-author. His main topic of interest appears to be nutrition and health.

2. A synopsis of the proposal

<u>For Operating Grants:</u> Identify the PURPOSE of the proposal, the HYPOTHESIS to be tested, or the QUESTIONS to be answered, the OBJECTIVES to be achieved, the APPROACH proposed, and the PROGRESS made to date.

<u>For Special Instrumentation or Equipment Requests:</u> Comment on the proposed uses, appropriateness and availability of similar equipment or services to the applicant's laboratory.

The study will evaluate the association between milk and dairy food consumption and growth and development indicators among children aged 5-11 years (n=158).

The stated objectives are: 1) to determine knowledge, attitude, and practice of dairy product consumption, as well as facilitators and barriers to dairy consumption among Canadian children over time; 2) to assess the contribution of milk and dairy products to total dietary intake and diet quality of children aged 5-11 years; and 3) to determine the association between milk and dairy consumption with body composition, cognitive development and indicators of growth and development in Canadian boys and girls aged 5-11 years.

The participants will be assessed every 6 months for 2.5 years thus the ages of the sample will be 7.5 to 13.5 years at the completion of the study. The design includes quantitative components to assess dietary intake, physical activity, growth (both from linear dimensions and IGF-1 status), body composition, cognitive development, and knowledge, attitude and practice toward milk and dairy food.

Qualitative components will assess the facilitators and barriers to milk and dairy food consumption through interviews with caregivers.

3. An assessment of the proposal

Discuss the strengths and weaknesses of the project. Assess the scientific merit of potential of the work proposed. State your recommendation, give clear and concise justification.

The stated title of the project is “the role of dairy in optimal growth and development in children and dairy protein”. Such a title is problematic in that it is difficult to establish what “optimal growth” is and how it can be defined and assessed in the context of this study. Questions such as whether optimal growth is associated with a specific growth rate and morphological profile, a specific body composition, and/or an absence if illness and thus good health need to be raised and substantiated. The fact that the objectives of the Special Call are primarily concerned with the role of dairy in the “optimal growth and development of children” and the role of dairy protein foods within a healthy plant-based diet and especially in relation to plant-based protein foods begs the question of whether “optimal growth” has previously been defined for this research grant call and if so, how does it relate to the proposed research. I could find no practical definition of optimal growth in the proposal, and it does not address this important outcome.

The design involves a longitudinal mixed-method study using both quantitative and qualitative assessment procedures. Specifically, the researchers propose to document milk and dairy product intakes of 5- to 11-year-old children every 6 months, over a 2.5-year period. This means that the final ages of the participants will be 7.5 to 13.5 yrs and data will be available from 5 years onwards. I am concerned that the researchers repeatedly state that information will be obtained on children aged 5 to 11 years when this is clearly not the case – the children will be 5.0 to 13.5 years of age and thus include both pre-adolescent and adolescent components. Given that the stated objectives of the study relate to children aged 5 to 11 years I am concerned that the proposers have not clearly thought through the adolescent component and its associated pubertal changes that will have a significant effect on knowledge and attitude towards nutrition and dietary intake. The only measure of puberty/adolescent maturity being proposed is to estimate the age at peak height velocity using a well-established procedure. However, this is a predictive measure and intrinsically carries error. It is highly likely that by 12 to 13 years of age 50% of the girls will have experienced menarche and 50% of the boys may well have experienced voice ‘breaking’ putting them into an advanced pubertal group in comparison to their early pubertal and pre-pubertal peers.

The proposal doesn’t address this potential problem.

I wonder if it would be more fruitful in this study to base the research questions regarding knowledge and attitudes to subgroups defined by pubertal status. In this regard I am not convinced that an assessment of IGF-1status is pertinent. Diurnal variation in IGF-1 is significant in children and some control of the timing of salivary sampling would be required. The proposal doesn't address this potential problem.

The measure of diet in children and adolescents is notoriously difficult. This is confounded by caregivers not having a complete picture of the dietary intakes whilst at school or in other social settings. This is particularly true for the intake of snacks and other foods eaten between meals which children and adolescents are unlikely to record or report with any degree of precision. Thus the 24-hour recalls completed by caregivers are highly unlikely to return a valid picture of dietary intake and may well exclude snacks and items not viewed favourably by care givers. The proposal doesn't address this potential problem.

Whilst the research team appear to have a good track record for studies of growth and development this proposal lacks the sort of detail that allows me to judge whether, in this instance, they have grasped the full research logistical implications of the timeline they propose. I think however, that it is a strong proposal in terms of its objectives but the numerous weaknesses with regard to design prevent me from rating it higher than "very good".

The Internal Reviewer's Report should include:

1. An assessment of the Principal Investigator:

Hassan Vatanparast (PI) is a professor in the field of nutritional epidemiology at the College of Pharmacy and Nutrition and School of Public Health, University of Saskatchewan. He has experience in conducting longitudinal studies and randomized controlled trials in community settings. His research experience focuses on evaluating the role of nutrition and lifestyle factors on growth and development of children in these studies. He is well funded and a productive researcher.

Jess Haines is the other lead and is a Professor of Applied Nutrition at the University of Guelph and the Co-Director of the Guelph Family Health Study, a family-based cohort study. She will be the major professor on the qualitative part of the study

Marta Erlandson is an Assistant Professor in the College of Kinesiology at the University of Saskatchewan. She is a certified exercise physiologist and a specialist in body composition assessment.

Dr. Walter Siqueira from College of Dentistry, University of Saskatchewan is an expert in salivary diagnostics

<u>Others- less defined role</u>
Adam Baxter Jones is a Kinesiology Professor and an associate member of the Department of Pediatrics – his specific role was not well defined but one assumes it will be around the physical activity

Ginny Lane is Assistant Professor of Nutrition at the University of Idaho and a registered dietitian- her expertise is in assesses food security of individuals. Role in this grant not entirely clear

Susan Petryk is an early career researcher with clinical experience that has helped her build clinically relevant patient-oriented research questions

2. A synopsis of the proposal:

To investigate how milk and dairy consumption is associated with growth and development indicators of 158 Canadian boys and girls aged 5-11 years.

Objectives
1) to determine knowledge, attitude, and practice of dairy product consumption, as well as facilitators and barriers to dairy consumption among Canadian children over time;
2) to assess the contribution of milk and dairy products to total dietary intake and diet quality in children aged 5-11 years; and

3) to determine the association between milk and dairy consumption with body composition, cognitive development and indicators of growth and development in Canadian boys and girls aged 5-11 years

They are proposing to use a longitudinal explanatory sequential mixed-method design to comprehensively evaluate the association between milk and dairy food consumption and growth, developmental and attitudes indicators. There is a quantitative and qualitative component of this

In the quantitative:

- will assess dietary intake (includes the contribution of dairy to dietary intake using serial 24hr recalls and a Food Frequency Screener that assesses the frequency of milk and dairy food consumption in the last six months and will include varying skin pigmentation (important for UV absorption and vitamin D synthesis), physical activity (Physical Activity Questionnaire for Children (PAQ-C)), sociodemographic and socioeconomic status, anthropometrics, and cognitive development, as well as bone, fat, and muscle mass development indicators (Anthropometric measures).
- The knowledge, attitude and practice toward milk and dairy food will also be collected from children and their caregivers (use the Knowledge Attitude and Practice (KAP) survey)
- a salivary sample will be taken to measure insulin-like growth factor-1, a growth marker. They state that they will be the first to do this
- Cognitive development

In the qualitative:

- to determine the facilitators and barriers to milk and dairy food consumption- using semi-structured interviews with children's main caregivers. The aim is to provide a more in-depth understanding of knowledge, attitudes, facilitators, and barriers regarding milk and dairy food consumption in Canadian children. Analysis of the interviews will be conducted using a thematic content analysis approach using the program NVivo.

The study will follow theoretical frameworks, including the component-cause model, life course theory and systems theory.

We hypothesize that appropriate intake of milk and dairy products has a positive association with growth and development parameters in children aged 5-11 years old.

Our proposed research aims to provide comprehensive information about the role of milk and dairy food consumption in growth and development indicators of Canadian children in a novel mixed-method longitudinal design

3. An assessment of the proposal:

strengths

- consistent with DFC call for this grant
- well established PI and co-PI.
- multi-disciplinary team brought together. Could have expanded the description of their roles in the trial a little more as the team is large
- good description of the methodologies proposed and reasonable justification for each
- KT section was well written- including the Partner Engagement and how this was included in all steps of the study.

Limitations

- some grantsmanship issues:
  - milestones not well written but table 2 is much better in explaining the study
  - objectives are written in different orders through the grant- gets a little confusing when trying to identify the primary outcome that the power calculation was based on. Later it is more clear that growth was the key outcome
- rational for cognitive assessment could have been stronger

Vatanparast

The role of dairy food consumption in optimal growth and development of Canadian children: A longitudinal mixed-method study

Applicants

Dr. Vatanparast is a Full Professor in the College of Pharmacy and Nutrition at the University of Saskatchewan. His research focuses broadly on nutrition, chronic disease and more recently, Indigenous and international health. He is well-funded with a number of grants currently held as PI, and he has previously held DFC funding. Dr. Vatanparast is well published, with 116 papers since 2014, with publications in areas relevant to the current proposal. He has very good supervisory experience.

Dr. Vatanparast is supported in this application by a number of experienced co-investigators with expertise in longitudinal childhood growth (Baxter Jones), musculoskeletal health and body composition (Erlandson), family-level factors and dietary intake among children and adolescents (Haines), newcomer children's nutritional health (Lane), developmental pediatrics (Petryk), and salivary diagnostics (Siqueira).

Synopsis

This proposal is predicated on mixed results in the current literature regarding the relationship between dairy intake and body composition in childhood, some of which may be due to the use of cross-sectional designs. The applicants therefore believe that longitudinal studies are warranted to clarify the relationship between milk and dairy product intake and body composition in children. Also, the applicants point out that there is scarce information on knowledge, attitudes, facilitators and barriers regarding milk and dairy food consumption among Canadian children aged 5-11 years.

On this background, the objectives of the current study are:

> **1)** to determine knowledge, attitudes, and practices of dairy product consumption, as well as facilitators and barriers to dairy consumption among Canadian children over time;
> **2)** to assess the contribution of milk and dairy products to total dietary intake and diet quality of children aged 5-11 years; and
> **3)** to determine the association between milk and dairy consumption with body composition (BMD, muscle and fat mass), cognitive development and indicators of growth and development in Canadian boys and girls aged 5-11 years.

The applicants propose to use a longitudinal explanatory sequential mixed-method design (in other parts of the grant this is referred to as a "contextually longitudinal explanatory sequential mixed-method design") to comprehensively evaluate the association between milk and dairy food consumption and growth and development indicators among 158 children aged 5-11 years followed for 2.5 years, with measurements collected every six months (total visits = 6) using quantitative and qualitative approaches. The applicants propose to enroll a sample of children that reflects the ethnic diversity of Saskatoon, including newcomer children.

Measurements of the children will include:

- dietary intake (three 24-h recalls every six months (six time-points) plus the FFQ from the CCHS), food security, physical activity, sociodemographic and socioeconomic status, anthropometrics, and cognitive development, as well as bone, fat, and muscle mass development indicators (using DXA); also, a salivary sample will be taken to measure insulin-like growth factor-1, a growth marker. Cognitive measures include the Canadian Wechsler Intelligence Scale for Children, version five;

- Knowledge, attitude and practice toward milk and dairy food will also be collected from children and their caregivers in the quantitative component.

- In the qualitative component, semi-structured interviews will be conducted with children's main caregivers to determine the facilitators and barriers to milk and dairy food consumption.

- The applicants indicate that proposed study will be informed by a combination of the following theoretical frameworks, including the component-cause model, life course theory and systems theory;

Assessment

Strengths

1. The PI is an active and motivated researcher who collaborates widely. He has obtained grants, published papers and led projects in areas relevant to this proposal;
2. The PI has assembled a highly capable group of co-I's for this project;
3. The application addresses important areas of research and is generally relevant to the call for proposals;
4. The synopsis at end of Background section (pg 5) summarizes the Background and encapsulates the key knowledge gaps in the current literature;
5. Many of the methods and / or equipment required for the proposed work are already established (or in place) in the applicants' centres;

Limitations

1. Pg 3 – In the background section on facilitators and barriers towards milk and dairy consumption, one prior study from Ontario is cited … is this the only study on this topic that has been done?

2. Methods, Pg 6 – The applicants indicate that the study will be informed by several theoretical frameworks, including the component-cause model, life course theory and systems theory. The applicants point out that these theoretical frameworks will assist in interpreting the results of the proposed study. It was not clear to me as I read the application how these 3 theoretical frameworks will be formally assessed or implemented at the analysis and interpretation stage;

3. Methods, Pg 7 – the sample size calculation is confusing as it seems to be predicated on being able to detect a significant change in growth. That children will grow over time is a given. In contrast, the objectives of the proposal are focused on longitudinal associations of variation (differences) in dairy product intake with differences in growth, cognition and other measures. Those important parameters (e.g. expected differences in outcomes according to differences in dairy intake) are not considered in the calculation. The total proposed sample size seems small as a consequence … 126 Canadian Children and 25 newcomer children … and it is not clear to me that the study will be adequately powered to detect important differences;

4. Methods, Pg 9 – While the proposed cognitive assessments are very interesting, it was not clear from the application whether children as young as 5 will sit down for a 2 hr self-administered cognitive assessment. Also, as this is a longitudinal study, will change in this outcome be measured and if so, what is the sensitivity to change of this instrument?

5. Methods, Pg 9 – bottom – qualitative component. How many participants will be involved?

6. Methods, Pg 10 – analysis – quantitative – it is not clear why a posteriori diet pattern analysis needs to be used to identify dairy intake patterns;

7. Methods, Pg 10 – analysis – quantitative – linear growth and adiposity are complex traits in the 5-11 year age window. Analytical challenges include non-linearity (growth spurts, adiposity rebound) and repeated measures. The applicants propose to random-effect multilevel modeling methods to analyze their data, although this important aspect of the analysis is described in only one sentence, and the application would have benefitted from additional details describing this aspect of the statistical analysis in light of these complexities;

8. Methods, Pg 11 –The "triangulation" section at the top of this page was difficult to follow. It is stated here that "the qualitative results will inform appropriate modeling with quantitative data, in effect 'following the thread'". Is it possible that themes will arise in the qualitative data that do not overlap with what is being collected in the quantitative portion?
